# Supplementary figures and images for: Breed-Related Differential microRNA Expression and Analysis of Colostrum and Mature Milk Exosomes in Bamei and Landrace Pigs
Source: Int J Mol Sci. 2024 Jan 4;25(1):667. doi: 10.3390/ijms25010667 (PMC10779168; doi:10.3390/ijms25010667)

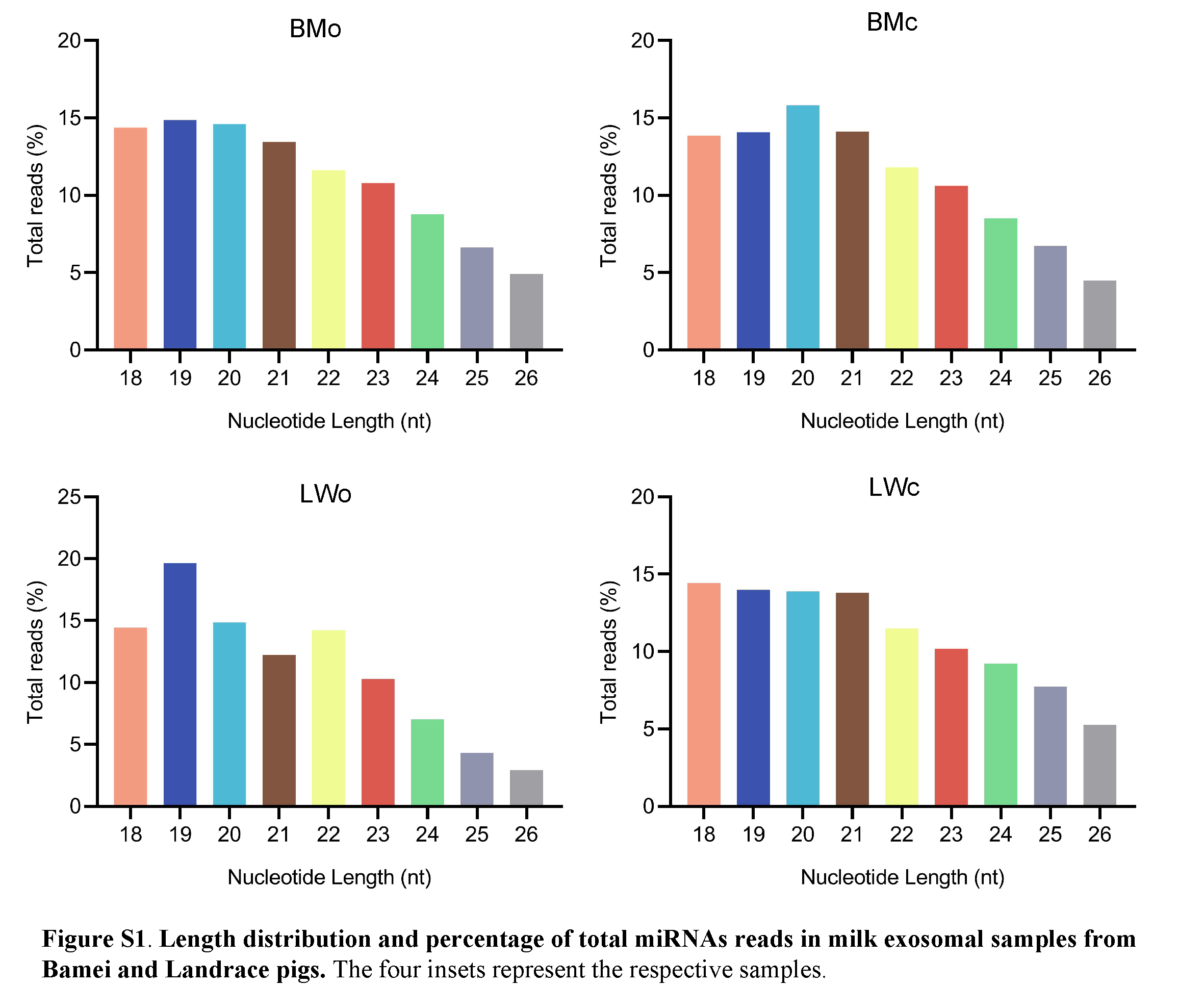

Supplement: Supplementary file 1 [file ijms-25-00667-s001.zip › Figure S1.tif]

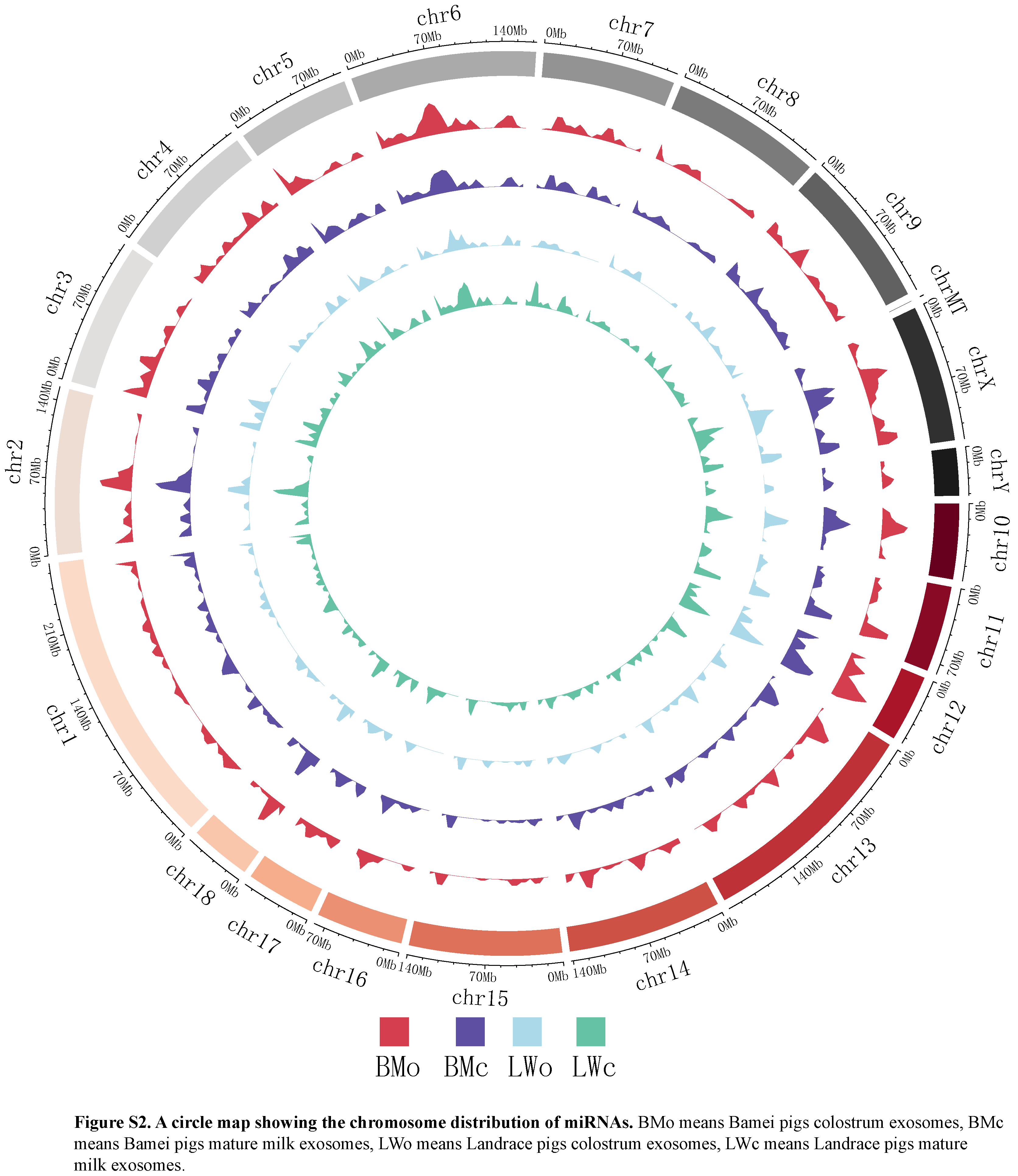

Supplement: Supplementary file 1 [file ijms-25-00667-s001.zip › Figure S2.tif]
